# Ceria Nanoparticles Decrease UVA-Induced Fibroblast Death Through Cell Redox Regulation Leading to Cell Survival, Migration and Proliferation

**DOI:** 10.3389/fbioe.2020.577557

**Published:** 2020-09-25

**Authors:** Fabianne Martins Ribeiro, Mariana Maciel de Oliveira, Sushant Singh, Tamil S. Sakthivel, Craig J. Neal, Sudipta Seal, Tânia Ueda-Nakamura, Sueli de Oliveira Silva Lautenschlager, Celso Vataru Nakamura

**Affiliations:** ^1^Programa de Pós-Graduação em Ciências Biológicas, Universidade Estadual de Maringá, Maringá, Brazil; ^2^Programa de Pós-Graduação em Ciências Farmacêuticas, Universidade Estadual de Maringá, Maringá, Brazil; ^3^Advanced Materials Processing and Analysis Center, Nanoscience Technology Center, University of Central Florida, Orlando, FL, United States

**Keywords:** antioxidant, photoaging, nanoceria, ultraviolet radiation, wound healing

## Abstract

Exposure to ultraviolet radiation is a major contributor to premature skin aging and carcinogenesis, which is mainly driven by overproduction of reactive oxygen species (ROS). There is growing interest for research on new strategies that address photoaging prevention, such as the use of nanomaterials. Cerium oxide nanoparticles (nanoceria) show enzyme-like activity in scavenging ROS. Herein, our goal was to study whether under ultraviolet A rays (UVA)-induced oxidative redox imbalance, a low dose of nanoceria induces protective effects on cell survival, migration, and proliferation. Fibroblasts cells (L929) were pretreated with nanoceria (100 nM) and exposed to UVA radiation. Pretreatment of cells with nanoceria showed negligible cytotoxicity and protected cells from UVA-induced death. Nanoceria also inhibited ROS production immediately after irradiation and for up to 48 h and restored the superoxide dismutase (SOD) activity and GSH level. Additionally, the nanoceria pretreatment prevented apoptosis by decreasing Caspase 3/7 levels and the loss of mitochondrial membrane potential. Nanoceria significantly improved the cell survival migration and increased proliferation, over a 5 days period, as compared with UVA-irradiated cells, in wound healing assay. Furthermore, it was observed that nanoceria decreased cellular aging and ERK 1/2 phosphorylation. Our study suggests that nanoceria might be a potential ally to endogenous, antioxidant enzymes, and enhancing the redox potentials to fight against UVA-induced photodamage and consequently modulating the cells survival, migration, and proliferation.

## Introduction

Nanotechnology has attracted tremendous attention in different fields of science including medicine and pharmacology. Special attention has been paid to the development of nanoparticles (NPs) demonstrating enzyme-like activities, referred to as nanozymes (Singh, 2019). Mimicking natural enzymes, nanozymes offer several advantages such as low cost, higher stability, and better catalytic efficiency (Wang et al., 2018; Singh, 2019).

Cerium oxide nanoparticles (CNPs or nanoceria) are particularly interesting nanomaterials in a wide range of biomedical applications, due to their antioxidant properties. Several studies support CNPs cytoprotective efficacy; demonstrating that different CNP formulations reduce chronic inflammation, promote angiogenesis, promote tissue regeneration, decrease cell death, and increase cell survival in model biological systems (Chigurupati et al., 2012; Das et al., 2014). Our group recently demonstrated that CNP also prevents UVB-induced fibroblast oxidative damage by decreasing reactive oxygen species (ROS) level and increasing antioxidant enzymes activities (Peloi et al., 2020). Cerium exists in a mixed valence state (Ce^3+^ and Ce^4+^) as an oxide and, due to the relatively low redox potential between these states, allows CNPs to have self-regenerative redox cycling properties, upon release or uptake of oxygen (termed *oxygen buffering* or *oxygen storage* capacity). This redox cycling, and interaction with the surrounding chemical environment, is evidenced practically as catalase (CAT) and superoxide dismutase (SOD)-mimetic activities. These surface reactions are catalytic and, thereby, remain active for an extended time protecting cells against the harmful effects of excess ROS production (Celardo et al., 2011; Singh et al., 2011; Das et al., 2014). Further, other studies have demonstrated indirect effects of nanoceria treatment on ROS levels as modulations in native antioxidant enzyme concentrations (e.g., SOD2, glutathione) (Das et al., 2018) as well as expression of proteins related to cellular oxygen metabolism (e.g., HIF1α) (Das et al., 2012).

Ultraviolet radiation (UV) is a well-known ROS inducer in human skin, contributing to the development of several chronic diseases and aging processes (Rinnerthaler et al., 2015). The effects of ultraviolet A rays (UVA, 320–400 nm) are well-recognized as being responsible for driving skin cells to senescence through the ROS-induced damage of essential cell macromolecules, including lipids, proteins, and nucleic acids. Modification of these species alters antioxidant cellular defense systems and disregulates important cell-signaling pathways (Krutmann and Schroeder, 2009) in deep skin layers, mainly affecting fibroblasts (Krutmann and Schroeder, 2009). These cells are the major cell type in the dermis and play a pivotal role in skin physiology (Heather et al., 2018) contributing to extracellular-matrix (ECM) and collagen production (maintaining the skin’s structural integrity) and playing an important role in cutaneous wound healing process (Bainbridge et al., 2013). In recent years, numerous studies have been conducted on the role of fibroblasts in wound healing and how this process gets disrupted under UVA radiation (Heather et al., 2018; Liu et al., 2018; Chen et al., 2019).

Thus, our goal was to study the effect of CNPs on cell survival, migration, and proliferation of L929 fibroblast cultures, at a low dose under UVA-induced oxidative redox imbalance. The current study extends the findings of another study on the photo-protective effects of nanoceria toward fibroblasts and keratinocytes (Caputo et al., 2015; Li et al., 2019). We believe that besides determining cell survival, CNPs can influence/preserve fibroblast migration and proliferation activities. Further, we investigate the efficacy of a higher Ce^3+^-containing formulation in producing these effects, in comparison to the higher Ce^4+^ formulation studied formerly. Our data showed that CNPs decrease UVA-induced fibroblast death through cell redox restoration leading to the modulation of signal-regulated protein kinases 1 and 2 (ERK 1/2) that control cells survival and proliferation. Additionally, we demonstrate improved proliferation and migration, following irradiation, *in vitro*, in nanomolar concentrations.

## Materials and Methods

### CNP Synthesis and Characterization

Cerium oxide nanoparticles were synthesized at 5 mM concentration via a wet chemistry approach producing particles in a size range of 3–5 nm, as described in our earlier publication (Pulido-reyes et al., 2015). In brief, a quantified amount of cerium nitrate hexahydrate salt (99.999% purity; Sigma-Aldrich) was dissolved in ultrapure (>20 MΩ) DI water and oxidized through addition of excess hydrogen peroxide solution (H_2_O_2_; from 3% stock solution, Sigma-Aldrich).

Particle suspensions were characterized, as previously described, and demonstrated similar character as noted in earlier investigations (Pulido-reyes et al., 2015). Particle morphology and size dispersion were determined using High Resolution Transmission Electron Microscopy (HRTEM; Philips Tecnai operating at 300 kV). Hydrodynamic radius (via dynamic light scattering) and surface charge (zeta potential) were obtained using a zeta sizer (Nano-ZS from Malvern Instruments). Optical spectra of these samples were characterized using a UV-visible spectrophotometer (PerkinElmer Lambda 7505). X-ray photoelectron spectroscopy (XPS) was carried out using Thermo Fisher ESCALAB 250xi spectrometer with a monochromatic Al Kα X-ray source.

### Cell Culture, Treatment, and UVA Irradiation

The experiments were conducted using the mouse fibroblast cell line L929 (ATCC^®^ CCL1^TM^, Manassas, VA, United States) cultured in DMEM (Dulbecco’s modified Eagle’s medium, Life Technologies/Gibco Laboratories, Grand Island, NY, United States) containing 10.0% fetal bovine serum (FBS, Life Technologies/Gibco Laboratories, Grand Island, NY, United States), 2 mM L-glutamine, supplemented with 10% (v/v) FBS and 1% (v/v) antibiotic solution (100 UI/mL penicillin and 100 μg/mL streptomycin) and incubated at 37°C in a 5% CO_2_ atmosphere. For all experiments, L929 cells were seeded at a density of 2.5 × 10^5^ cells/mL and were below 20 passages.

For cytotoxicity, photoprotection and mitochondrial membrane potential (ΔΨm) measurement assays, cells were seeded into 96-well plates. For wound healing, cell growth, β-galactosidase (SA-βG), and cell death assays, cells were seeded into 24-well plate. For other experiments, cells were seeded into 6-well plates.

For the treatment procedure, cell monolayers were washed three times with phosphate-buffered saline buffer (PBS) and pre-treated with 100 nM CNP diluted in serum-free DMEM for 24 h at 37°C in a humidified atmosphere with 5% CO_2_, followed by irradiation. Other studies have noted the utility of producing an intra-cellular nanoceria population in conferring strong radio-protection. Given the significant radical scavenging activity of nanoceria, it is reasonable that the particles largely protect cells from the indirect effects of radiation: necessitating pre-incubation/prophylactic treatment to allow for appreciable cellular uptake. Thus, pre-treatment was chosen over co- or post-treatment for the present study.

For irradiation procedure, cells were washed with PBS and Hank’s balanced salt solution supplemented with glucose (HBSS, Sigma-Aldrich, St. Louis, MO, United States) followed by irradiation at intermittent dose of 15 J/cm^2^ or with a unique dose of 30 J/cm^2^ via UVA lamps (Philips TLK 40 W/10R lamp, Netherlands), for 40 and 80 min, respectively, set up at a distance of 20 cm from the plates and monitored using a radiometer sensor (peak: 365 nm, VLX-3W, Vilber Lourmat, Marne La Vallée, France). After irradiation, HBSS was changed by DMEM serum-free and cells were immediately assayed or maintained in an incubator at 37°C in a humidified atmosphere with 5% CO_2_ for the time required for each assay. N-acetylcysteine (NAC, 100 μM^–1^ h treatment, Sigma-Aldrich, St. Louis, MO, United States) was used as antioxidant control.

### Cytotoxicity and Photoprotection Assay

To assess the cytotoxic potential of CNPs we employed the MTT assay. Briefly, L929 cells were treated with samples in various concentrations of 500, 100, 50, 10, and 5 nM for 24 h at 37°C in a humidified 5% CO_2_ incubator. After, cells were washed with PBS and 50 μL of MTT (2 mg/mL) was added followed by incubation for 4 h. The medium was removed and cells were washed followed by DMSO addition. Absorbance at 570 nm was determined (BioTek, PowerWave XS microplate spectrophotometer) and the percentage of viable cells was determined relative to the untreated control group.

To assess UVA phototoxicity, L929 cells were treated with 100, 50, 10, and 5 nM CNP for 24 h. After, the CNP solution was replaced with HBSS and cells were irradiated with UVA (30 J/cm^2^) and then incubated with serum-free DMEM for 24 h. After, cytotoxicity was detected by MTT assay.

### Measurement of Intracellular ROS

Intracellular ROS was measured using 2′,7′-dichlorodihydrofluorescein diacetate (H_2_DCF-DA, Eugene, OR, United States). Briefly, L929 cells were treated with 100 nM CNP for 24 h or 100 μM NAC, 100 UI SOD (Sigma-Aldrich, St. Louis, MO, United States) and 100 UI catalase (CAT, Sigma-Aldrich, St. Louis, MO, United States) for 1 h and irradiated with UVA at a unique dose of 30 J/cm^2^ or with 15 J/cm^2^ followed by an additional dose (15 J/cm^2^) after 24 h. After different times, cells were incubated with 5 μM H_2_DCF-DA for 30 min in the dark at 37°C. Cell-associated fluorescence was detected using a spectrofluorimeter (VICTOR X3, PerkinElmer, United States, λex = 488 nm, λem = 525 nm). The fluorescence percentage was expressed as arbitrary fluorescence units per μg of protein determined by Bradford method (Bio-Rad, CA, United States).

### Intracellular Antioxidant Enzymes Activity Measurements

To evaluate intracellular antioxidant enzymes activity, after 100 nM CNP or 100 μM NAC treatment, L929 cells were irradiated with UVA (30 J/cm^2^) and incubated for 1 h. Cells were resuspended and lysed in cold Tris buffer (Tris 10 mM, pH 7.4), sonicated for 60 s with a 30% pulse. The cell debris was removed by centrifugation at 14,000 rpm for 10 min at 4°C and the supernatants were assayed for GSH levels and SOD activity. Protein concentration was determined by Bradford method.

To measure GSH levels *o*-phthalaldehyde (OPT) was used. Cell lysate supernatant (50 μg/mL of protein) was transferred to a black 96-well microplate containing sodium phosphate buffer (100 mM KH_2_PO_4_–KOH, pH 10, 185 μL) followed by 10 μL OPT (10 mg/mL in ice cold methanol) addition. After 25 min of incubation in the dark with gentle mixing, the plate was read in a fluorescence plate reader (VICTOR X3, PerkinElmer, United States, λex = 350 nm, λem = 420 nm) (Schaffer et al., 2011).

The enzymatic SOD activity was determined by measuring the inhibition of pyrogallol autoxidation. In brief, 930 μL of Tris buffer (200 mM Tris, 2 mM EDTA, pH 8.2) and 50 μg of cell lysate protein were mixed followed by addition of 70 μL pyrogallol solution (15 mM in 1 mM Tris–HCl, pH 8.2) and the absorbance was read at 420 nm (Shimadzu, UV-1700). One unit of SOD activity was considered based on 50% of the pyrogallol oxidation (expressed as unit of SOD/μg protein).

### Cell Growth Assay

The L929 cells were treated with 100 nM of CNP for 24 h or 10 μM of NAC for 1 h. After, the medium was harvested and cells were washed with PBS followed by irradiation with UVA (30 J/cm^2^). The cells were collected by trypsinization right after irradiation or after incubation for 1, 2, 3, 4, and 5 days in DMEM supplemented with 1% FBS. The medium was not replaced from wells at each time point. The cells were manually counted using a Neubauer chamber by the trypan blue exclusion method.

### Wound Healing Assay

The L929 cells were treated with 100 nM of CNP for 24 h and irradiated with UVA (30 J/cm^2^). Immediately after irradiation, a sterile 200 μL pipette tip was used to make a straight scratch on the monolayer of cells attached. The pictures were taken at 0, 24, and 48 h after the scratch. Wound repopulation was assessed with a light microscope (Olympus BX51, Miami, FL, United States) equipped with a digital camera (Olympus C5060, Miami, FL, United States). Photomicrographs were taken at 5× magnification and cell proliferation area was measured using Image-J 1.45S software (Wayne Rasband, National Institutes of Health, Bethesda, MD, United States).

### Senescence-Associated β-Galactosidase Assay

The senescence-associated β-galactosidase (SA-βG) was performed as previously described (Yang and Hu, 2004). The L929 cells were treated with 100 nM CNP for 24 h or 100 μM NAC for 1 h. Next, the cells were irradiated with UVA (15 J/cm^2^) for three consecutive days and incubated for 24 h in a humidified incubator. Cells were washed in phosphate-buffered saline (PBS; pH 7.4) and fixed for 5 min in 2% formaldehyde and 0.2% glutaraldehyde in PBS. After, 100 μL of staining solution (citrate–phosphate buffer with 100 mM potassium ferricyanide, 100 mM potassium ferrocyanide, 5 M NaCl, 0.2 M MgCl_2_) was added followed by the addition of 10 μL of 2 mM Di-β-D-galactopyranoside (FDG, Molecular Probes, Eugene, OR, United States) per well. The plate was incubated at 37°C in the dark for 24 h. After, the supernatant (100 μL) was transferred to a 96-black-well plate in triplicates for fluorescent measurement using a spectrofluorometer (Victor X3; PerkinElmer; λex = 485 nm, λem = 535 nm). Doxorubicin (DOXO, 5 μg/mL), used as a positive control, was added to cells for 24 h followed by incubation of 3 days. The fluorescence percentage was expressed as arbitrary fluorescence units per μg of protein determined by Bradford method.

### Cell Death

Caspase-like activity was performed using an EnzChek Caspase-3 #1 Z-DEVD-AMC Substrate Assay Kit (Molecular Probes, Eugene, OR, United States). The L929 cells were treated with 100 nM CNP for 24 h or 100 μM NAC for 1 h. Next, the cells were irradiated with UVA (30 J/cm^2^). After, the cells were incubated for 24 h and after, collected, washed, resuspended in PBS buffer and processed according to the manufacturer’s instructions. The samples were then added to a black 96-well plate, and fluorescence was measured in a spectrofluorometer (Victor X3; PerkinElmer; λex = 342 nm, λem = 441 nm). Camptothecin (CAMP, 100 μM, 1 h treatment) was used as a positive control. An additional group was incubated with the caspase inhibitor Ac-DEVD-CHO. The fluorescence percentage was expressed as arbitrary fluorescence units per μg of protein determined by Bradford method.

### Mitochondrial Membrane Potential (ΔΨm) Measurement

We examined the mitochondrial membrane potential (ΔΨm) through tetramethyl rhodamine ethyl ester (TMRE, Molecular Probes, Eugene, OR, United States) labeling. In brief, L929 cells were treated with 100 nM CNP for 24 h or 100 μM NAC for 1 h and irradiated with UVA (30 J/cm^2^) followed by 2 h incubation. The cells were washed, harvested and resuspended in saline solution (0.9% sodium chloride solution), and 100 nM TMRE was added for 20 min in the dark at 37°C (Xuan et al., 2014). After, cells were washed, and cell-associated fluorescence was detected using a spectrofluorimeter plate reader (VICTOR X3, PerkinElmer, United States, λex = 549 nm, λem = 575 nm). Carbonyl cyanide 3-chlorophenylhydrazone (CCCP, 200 μM, 1 h treatment – Sigma-Aldrich, St. Louis, MO, United States) was used as a positive control. The fluorescence percentage was expressed as arbitrary fluorescence units per μg of protein determined by Bradford method.

### Expression and Quantification Analysis of ERK 1/2 Protein

Western blot was performed to detect ERK 1/2 protein. After treatment with 100 nM CNP for 24 h or 100 μM NAC for 1 h L929 cells were irradiated with UVA (30 J/cm^2^) followed by 2 h incubation. Next, cells were lysed in lysis buffer (1%) and total protein (20 μg) was separated by 12% sodium dodecyl sulfate polyacrylamide gel electrophoresis and transferred to 0.22-μm nitrocellulose membranes. The membranes were blocked with 5% albumin diluted in a Tris-buffered saline solution containing 1% Tween-20 (TBST) and then incubated overnight at 4°C in solutions with primary antibodies (1: 50) against Erk1/2 (sc-514302), phospho ERK1/2 (sc-81492), or PCNA (1: 10,000, sc-56) (Santa Cruz Biotechnology, Santa Cruz, CA, United States). The membranes were washed three times with TBST before an incubation for 1 h in solution with anti-mouse secondary antibody HRP-conjugated (1:10,000) (Santa Cruz Biotechnology, Santa Cruz, CA, United States). Proteins were detected by western blotting luminol reagent (Santa Cruz Biotechnology, Santa Cruz, CA, United States) using CCD camera imaging system (ImageQuant LAS 500, GE Healthcare Life Sciences, Uppsala, Sweden). Image-J 1.45S software (Wayne Rasband, National Institutes of Health, Bethesda, MD, United States). Quantitation of the relative amount of p-ERK 1/2 was normalized to the control PCNA.

### Statistical Analysis

All other experiments were performed in duplicate and repeated three times. The mean values were expressed as mean ± standard deviation (SD), followed by analysis of variance (ANOVA) and Tukey test (Prism 5.0 software). Values of *p* < 0.05 were considered statistically significant.

## Results

### Nanoceria Materials Characterization

**FIGURE 1:**
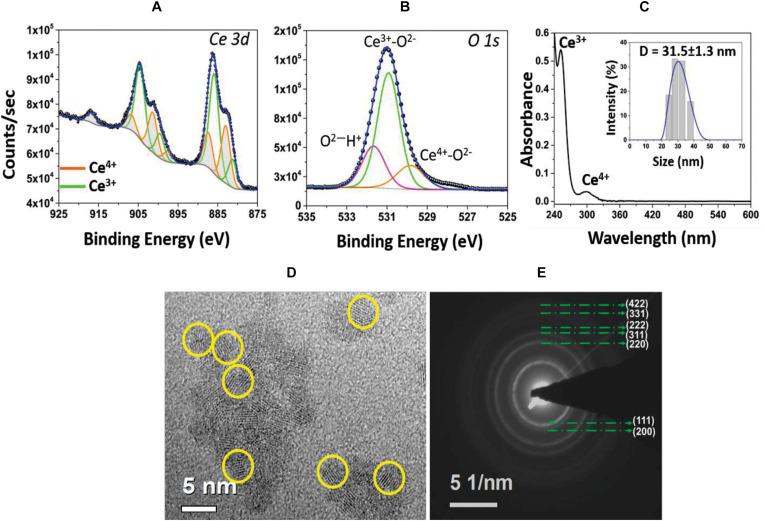
**(A)** High resolution XPS spectrum of Ce 3d envelope. Deconvoluted and peak fitted spectrum shows variations in the multiplet components of Ce 3d_5__/__2_ and Ce 3d_3__/__2_ doublets. The characteristic peaks of Ce^3+^ (green lines) and Ce^4+^ (orange lines) oxidation states are identified. **(B)** Asymmetry in the O1s spectrum and deconvoluted peaks are attributed to ions associated with the Ce^4+^ and Ce^3+^ as well as surface hydroxyl species, OH/O^2^. **(C)** UV-Visible spectrum of CNP sample evidencing signature peaks at 253 nm, corresponding to Ce^3+^ population, and at 300 nm, for Ce^4+^ oxidation state. Inset: hydrodynamic size (∼31.5 nm diameter) of CNPs with narrow size distribution. **(D)** HRTEM image of CNP with the particle size of 3–5 nm and **(E)** SAED pattern illustrating the material’s nano-crystalline character.

In order to investigate the surface chemistry of CNPs, Ce3d and O1s XPS spectra were collected (Figures 1A,B). Each element is plotted with fitted and deconvoluted peaks, along with the actual/experimental spectra. The Ce3d spectrum is comparatively complex due to the presence of both 3+ and 4+ oxidation states in the material and d-orbital, multiplet splitting. The spin-orbit doublet 3d_3__/__2_ (880.3 and 898.3 eV) and 3d_5__/__2_ (898.6 and 916.5 eV) is evident for both oxidation states of Ce. The peaks plotted in green are characteristic of the Ce^3+^ oxidation state, while the peaks plotted in orange are of Ce^4+^ (Seal et al., 2020). The percent of surface Ce^3+^ (or Ce^4+^) states in CNP was calculated from the ratio of the summed Ce 3d peak areas associated with Ce^3+^ (or Ce^4+^) to the total integral area for the whole Ce 3d region. From this analysis, it was found that the concentration of Ce^3+^ (58%) was higher than the Ce^4+^ (42%). In addition, the O1s spectrum of CNP was fitted to characterize the local environment around oxygen. Three peaks were recognized that belong to Ce^4+^-O^2–^, Ce^3+^-O^2–^, and O^2–^^–^H+. Additionally, it was found that the relative concentration of Ce^3+^-O^2–^ is higher than Ce^4+^-O^2–^, confirming the CNP sample is rich in Ce^3+^ valence state. Figure 1C shows the UV-Visible spectrum of CNP sample where the relative intensity of the signature peak at 253 nm indicates more Ce^3+^ oxidation state present in the material surface. The inset in Figure 1C shows the hydrodynamic diameter of the synthesized CNPs, found to be 31.5 ± 1.3 nm. The dry nanoparticle size from HRTEM was measured as ∼5 nm (Figure 1D), indicating that the particles are ultra-small in diameter with a substantial hydration layer. Diffraction rings observed in SAED pattern (Figure 1E) match the cubic structure of CeO_2_. In addition, the surface zeta potential of CNP was measured as 25.4 mV: indicating colloidal stability in water.

### CNP Induces Cytoprotective and Photoprotective Effect in UVA-Irradiated L929 Fibroblasts

**FIGURE 2:**
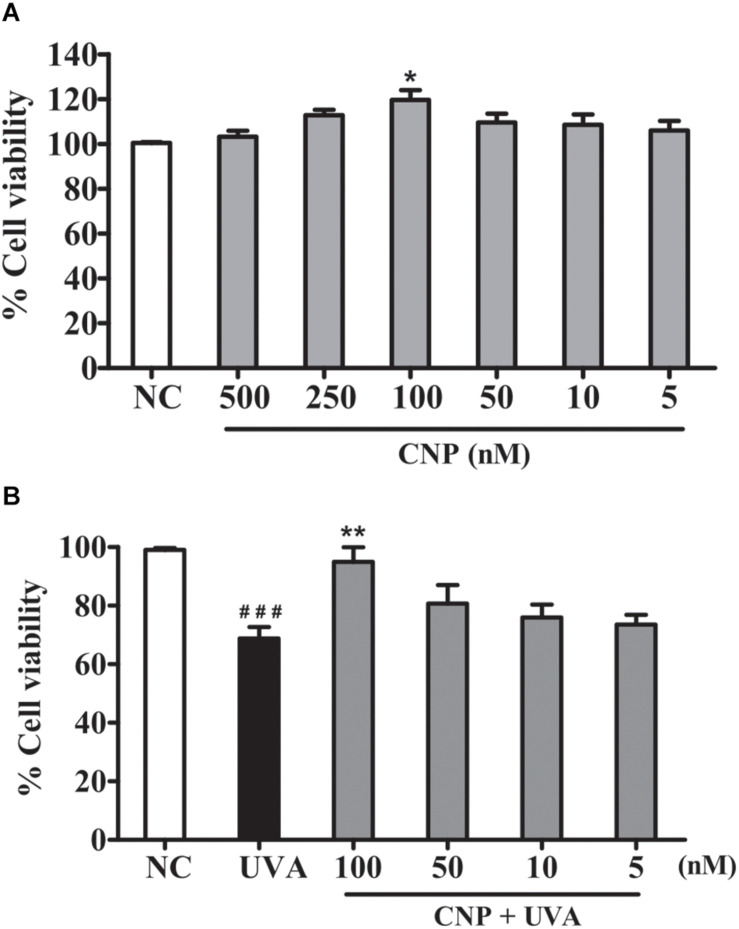
Effect of CNP on viability of non-irradiated and irrradiated L929 cells. **(A)** Cells were treated with CNP (500, 250, 100, 50, 25, and 5 nM) for 24 h. **(B)** Cells were treated with CNP (100 nM), exposed to UVA radiation (30 J/cm^2^) and incubated for more 24 h. In both experiments, cell viability was assessed by MTT assay. NC (non–treated and non-irradiated cells), CNP (treated and non-irradiated cells), UVA (non-treated and irradiated cells) and CNP + UVA (treated and irradiated cells). *p < 0.05, significantly different from NC; **p < 0.01, significantly different from UVA; ^###^*p* < 0.001, significantly different from NC.

Cell viability was assessed in fibroblast cell line L929 treated with CNP at different concentrations 500, 250, 100, 50, 10, and 5 nM using MTT assay. Figure 2A shows that CNP does not affect the viability of L929 cells compared with non-treated group (NC). Moreover, it was noted that 100 nM CNP significantly (*p* < 0.05) induces cell growth (15%) compared with control (non-treated cells). The photoprotective effect of CNP in L929 cells against UVA radiation was also evaluated. As shown in Figure 2B, after irradiation the cells treated with 100 nM CNP significantly increased (26%) cell viability compared with only UVA irradiated group. Based on this observation, 100 nM was chosen for our further investigations.

### CNP Decreases ROS Formation and Increases SOD Activity and GSH Level in UVA-Irradiated L929 Fibroblasts

**FIGURE 3:**
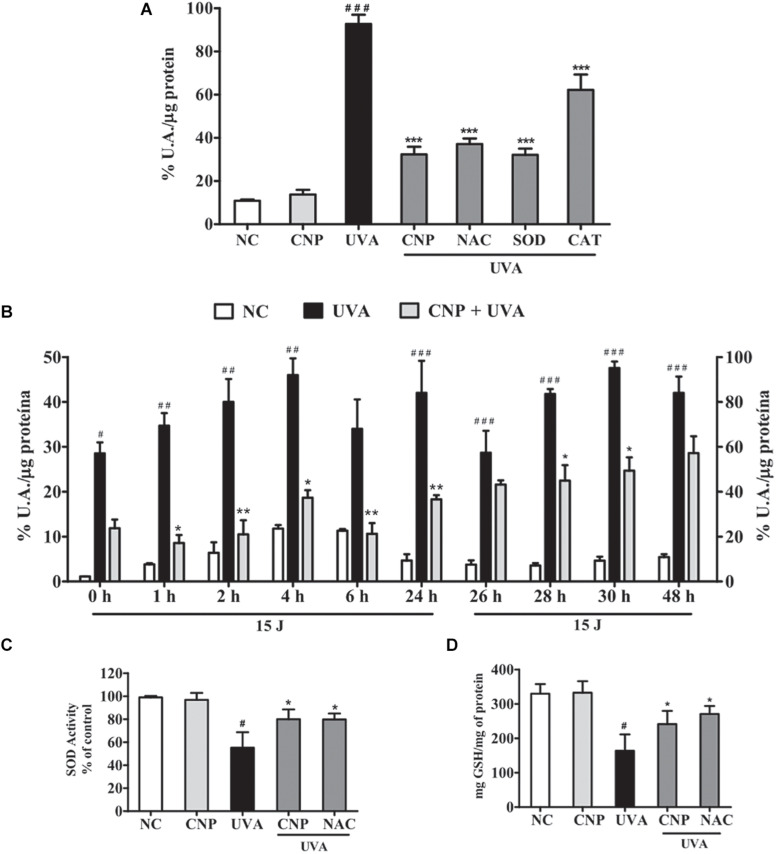
CNP antioxidant effect on UVA-irradiated L929 fibroblasts. **(A,B)** Detection of total ROS in L929 cells treated with CNP (100 nM) for 24 h and irradiated with UVA, using H_2_DCFDA. **(A)** Cells were exposed to UVA radiation (30 J/cm^2^) and the readings performed immediately after irradiation. **(B)** Cells were exposed to UVA radiation (15 J/cm^2^), and the readings performed in different times (0, 1, 2, 4, 6, and 24 h). After 24 h of incubation the cells were reirradiated with 15 J/cm^2^ and the readings performed in different times (26, 28, 30, and 48 h). The level of intracellular ROS is expressed as the percentage mean of DCF fluorescence intensity. **(C**) Detection of SOD activity and **(D)** GSH levels in L929 cells treated with CNP (100 nM) for 24 h and irradiated with UVA (30 J/cm^2^). The readings were performed after 1 h. SOD activity assessed by autoxidation of pyrogallol. GSH content was assayed by the o-phthalaldehyde method. NC (non-treated and non-irradiated cells), UVA (non-treated and irradiated cells), CNP + UVA (treated and irradiated cells), CNP (treated and non-irradiated cells), NAC + UVA (cells treated with NAC and irradiated). SOD + UVA (cells treated with superoxide dismutase and irradiated), CAT + UVA (cells treated with catalase and irradiated). *p < 0.05, significantly different from UVA; **p < 0.01, significantly different from UVA; ***p < 0.001, significantly different from UVA, ^#^*p* < 0.05, significantly different from NC, ^##^*p* < 0.01, significantly different from NC; ^###^*p* < 0.001, significantly different from NC.

The intracellular antioxidant activity of nanoceria on UVA-induced L929 oxidative stress was analyzed using H_2_DCF-DA. We observed that immediately after irradiation, CNP showed pronounced effect, reducing 60% of intracellular ROS formation, compared with UVA (Figure 3A). Similar effect (60%) was observed for SOD treatment (Figure 3A). NAC and CAT inhibited 55 and 30% of ROS, respectively, compared with UVA. A time course analysis (Figure 3B) showed that CNP effect persisted for 48 h. After 1 h of a single dose of UVA radiation, CNP significantly (26%) reduced ROS formation compared with UVA irradiation in absence of CNP treatment. This effect lasted until 24 h with significant ROS reduction up to 30% compared with UVA. After an additional dose of UVA irradiation, CNP was still able to reduce ROS production. This effect was again persistent for more than 24 h with a significant ROS reduction up to 46%. For the time 0, 24, and 48 h we can see that CNP decreased UVA-induced ROS production, however the differences observed were not significant.

Cerium oxide nanoparticle effect on L929 intracellular antioxidant enzymes SOD and GSH activity was also determined. Figures 3C,D show that the exposure of L929 cells to UVA significantly decreases SOD activity (55%) and GSH levels (50%) compared with non-treated and non-irradiated cells. The treatment with CNP led to a significant increase in the activity of SOD (25%) and GSH levels (47%) compared with UVA. Pretreatment with NAC also significantly increased SOD activity and GSH levels by 25 and 65%, respectively.

### CNP Induces L929 Cell Proliferation and Migration in UVA-Irradiated L929 Fibroblasts

**FIGURE 4:**
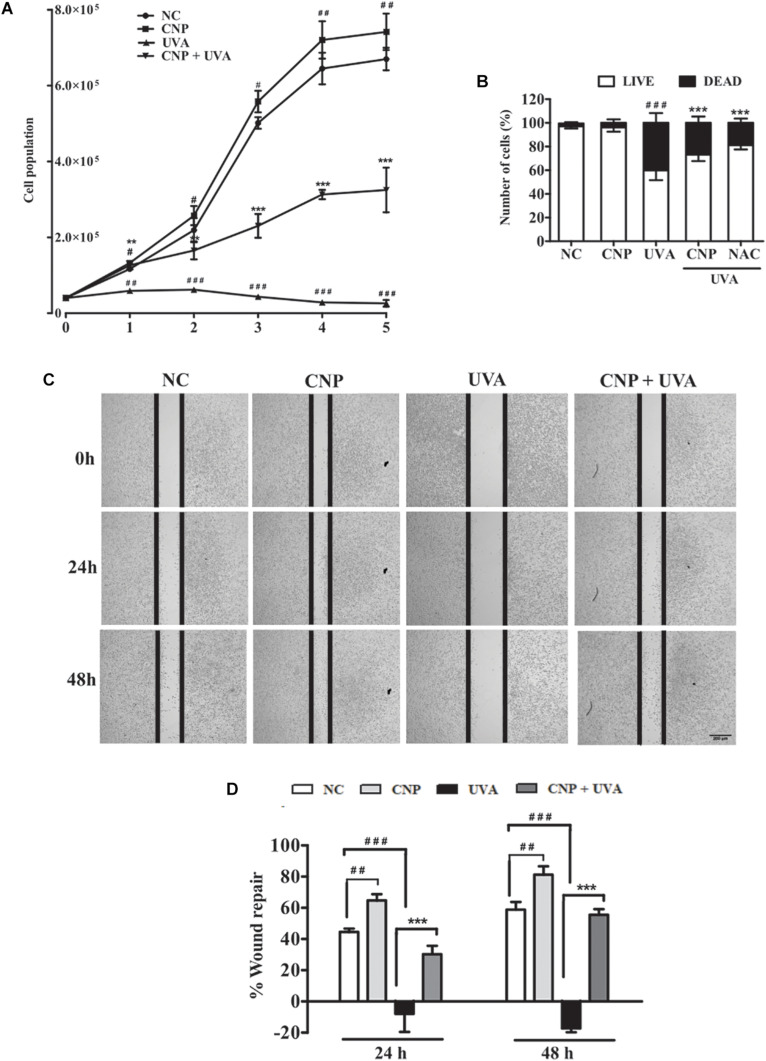
Evaluation of L929 cell proliferation and migration. **(A,B)** Effect of CNP on L929 cells proliferation. Cells were treated with CNP (100 nM) for 24 h followed by irradiation with UVA (30 J/cm^2^) and incubated for 1, 2, 3, 4 and 5 days. Every 24 h cells were counted using trypan blue dye exclusion method, for 5 days considering live cells. Dead and live cells were quantified after 24h incubation after irradiation (day 1) and counted by trypan blue dye exclusion method. **(C,D)** Wound healing assay. L929 cells were treated with 100 nM CNP for 24 h, irradiated with 30 J/cm^2^ UVA, and cells were scratched. Representative cell images from each group in the indicated time points after scratrch are shown. The area of the wound was measured at the 0, 24 and 48 h time points and compared in every group. Photomicrographs were taken at ×5 magnification in a light microscope. NC (non-treated and non-irradiated cells), UVA (non-treated and irradiated cells), CNP + UVA (treated and irradiated cells), CNP (treated and non-irradiated cells), NAC + UVA (cells treated with N-Acetylcysteine and irradiated). ^#^*p* < 0.05: significantly different from NC; ^##^*p* < 0.01: significantly different from NC, ^###^*p* < 0.001: significantly different from NC, **p < 0.01: significantly different from UVA; ***p < 0.001: significantly different from UVA.

To understand the possible ability of CNP on L929 cell proliferation, we performed a growth curve assay. In non-irradiated cells CNP increased about 12% cell growth in all tested days compared with NC (Figure 4A). CNP treatment also induced a progressive cell growth in 1, 2, 3, 4, and 5 days after radiation of 2.5, 5.1, 5.3, 11.0, and 15.0-fold, respectively, compared with UVA (Figure 4A). Cells were also counted 24 h after irradiation considering live and dead cells. As shown in Figure 4B, the dead cells in CNP irradiated cells was 22 and 14% in NAC treated cells compared with UVA.

In addition of CNP induced L929 cell growth, we evaluated the CNP L929 migration in wound repair. Wound recovery in cells under normal conditions was 44% at 24 h and 58% at 48 h compared with time zero (Figure 4C,D). The wound repair was significantly higher in cells treated with CNP than those in the control group. This increase was 64% and 81% after 24 and 48 h, respectively. No cell migration was observed in the UVA group, but CNP treatment promoted 30 and 55% of wound repair after 24 and 48 h of UVA irradiation, respectively.

### CNP Decrease Senescence and Apoptosis in UVA-Irradiated L929 Fibroblasts

**FIGURE 5:**
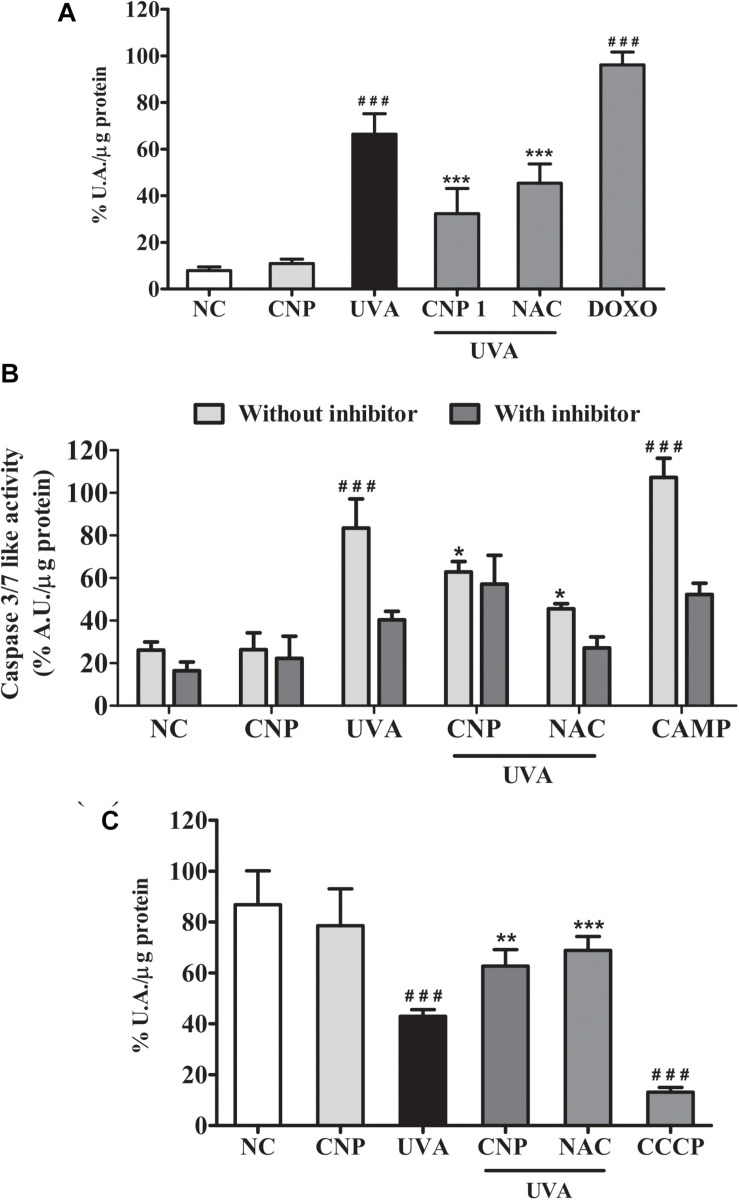
Assessment of senescence and cell death in L929 cells. **(A)** Senescence-associated β-galactosidase (SA-bG) activity measured based on fluorescein production. Cells were treated with 100 nM CNP and irradiated with to 10 J/cm^2^ for three consecutive days and incubated for 24 h. Fluorescence was measured after 24 h of incubation with FDG. **(B)** Caspases 3/7 like activity. Cells were treated with 100 nM CNP and irradiated with 30 mJ/cm^2^ UVA. Caspase-3/7 activity was measured using Z-DEVD-AMC substrate after 24 h of irradiation. **(C)** Measurement of mitochondrial dysfunction. Cells were treated with 100 nM CNP and irradiated with (30 J/cm^2^) After 2 h of incubation, cells were stained with the fluorescent probe (TMRE, 100 nM). NC (non-treated and non-irradiated cells), UVA (non-treated and irradiated cells), CNP + UVA (treated and irradiated cells), CNP (treated and non-irradiated cells), NAC + UVA (cells treated with N-acetylcysteine and irradiated), DOXO (cells treated with doxorubicin and non-irradiated), CAMP (cells treated with camptothecin and non-irradiated), CCCP (cells treated with carbonyl cyanide 3-chlorophenylhydrazone and non-irradiated. ^###^*p* < 0.001, significantly different from NC; ***p < 0.001, significantly different from UVA; **p < 0.01, significantly different from UVA; *p < 0.05, significantly different from UVA.

The effect of CNP in UVA-induced cellular senescence of L929 cells was also evaluated using fluorescein di-β-D-galactopyranoside (FDG). As shown in Figure 5A, we observed that UVA irradiation induced significant cell senescence (66%) after three days post-irradiation. β-galactosidase activity was not detected after a short period of analysis (less than 3 days after irradiation) (data not shown). CNP treatment significantly decreased (32%) β-galactosidase activity in irradiated cells, compared with UVA. A significant decrease in β-galactosidase activity was also observed for NAC (45%). DOXO, used as a positive control, increased 89% of β-galactosidase activity compared to NC.

The effect of CNP in UVA-induced apoptosis of L929 cells was assessed by measuring Caspase 3/7 levels. Figure 5B shows a significant increase in the activity of Caspase 3/7 (57%) in UVA irradiated L929 cells compared with NC. CNP treatment decreased by 25% the activity of Caspase 3/7 in irradiated cells, compared with UVA. For NAC this effect was higher (53%) than CNP. CAMP, used as a positive control, increased the activity of Caspase 3/7 like activity by 74%, compared with NC. As expected, the group incubated with the caspase inhibitor Ac-DEVD-CHO decreased the UVA (43%) and CAMP (55%) effect in Caspase 3/7 activity. Interestingly, CNP had a similar effect (25%) as observed for the caspase inhibitor on UVA-induced Caspase 3/7 activity.

Additional signals of apoptotic features were assayed to study mitochondrial dysfunction. Thus, the effect of CNP in mitochondrial membrane potential of UVA irradiated L929 cells was assessed using TMRE. Figure 5C shows significant mitochondrial membrane depolarization in UVA exposured cells (44%), compared with NC. CNP treatment protected cells by significantly decreasing 20% the mitochondria depolarization, compared with UVA. A similar effect was observed for NAC (26%).

### CNP Inhibits ERK Phosphorylation in UVA-Irradiated L929 Fibroblasts

**FIGURE 6:**
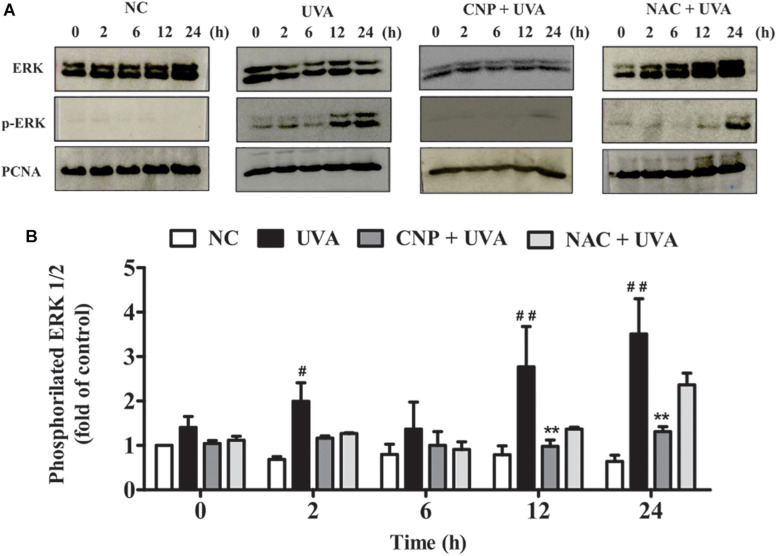
ERK phosphorylation and cell cycle analysis. **(A,B)** ERK 1/2 phosphorylation was performed by western blot. After 24 h of CNP (100 nM) treatment and UVA exposure (30 J/cm^2^) cell lysates were prepared for subsequent analysis by polyacrylamide gel electrophoresis followed by western blot analysis for ERK 1/2 (ERK total), phospho-ERK 1/2 (p-ERK 1/2) and PCNA. The density of each band was normalized with corresponding PCNA levels (bar graphs). NC (non-treated and non-irradiated cells), UVA (non-treated and irradiated cells), CNP + UVA (treated and irradiated cells), CNP (treated and non-irradiated cells), NAC + UVA (cells treated with N-acetylcysteine and irradiated). ^#^*p* < 0.05, significantly different from NC; **p < 0.01, significantly different from UVA; ^##^*p* < 0.01, significantly different from NC.

To assess ERK contribution to cell death, survival and/or proliferation of L929 cells under UVA radiation we performed immunoassay. As shown in Figures 6A,B, ERK phosphorylation in UVA group was significantly increased after 2 h (1.31-fold), 12 h (2.0-fold), and 24 h (2.9-fold) of irradiation compared with NC. In CNP treated cells ERK phosphorylation was significantly lower in 12 h (1.8-fold) and 24 h (2.2-fold) compared with UVA group. NAC used as an antioxidant control decreased UVA-induced ERK phosphorylation in 12 h (1.4-fold) and 24 h (1.2-fold) but this decrease was not significant.

## Discussion

Cerium oxide nanoparticles have attracted increasing interest for medical applications, due to their unique nanoscale characteristics which confer self-regenerative antioxidant capability (Scirè and Palmisano, 2020). CNP cytoprotective effect has been described, in a recently published work, for UVA-irradiated fibroblasts (Li et al., 2019). Further, the utility of CNPs in contributing both direct and indirect protection from irradiation damage has been demonstrated as UV-shielding and potent antioxidant action in preventing UV-induced cell damage and mutagenesis in Jurkat and HaCaT cells, respectively (Caputo et al., 2015). Here, our goal was to reveal whether under UVA-induced oxidative redox imbalance, a low dose of CNP (nanomolar concentration) induces extended cyto-protective effects on cell survival, senescence, migration, and proliferation. To this end, high doses of UVA were utilized and the changes to antioxidant/ROS activity and cell survival were monitored over several days, post-irradiation. The data obtained showed that pre-treatment with a low dose of CNP restores endogenous antioxidant enzymes activity which in turn prevents cell aging and induces a decrease in the activation of signaling pathways that control cell survive (i.e., attenuating activation of apoptotic pathway activation).

We first showed that CNP pre-treatment induced no L929 cytotoxicity, even at high concentrations (up to 500 μM) (data not shown). It should be noted that the CNP dose in this study was about 1,000 times lower than those used in previous studies (Caputo et al., 2015; Li et al., 2019). Other studies have demonstrated the value of CNP pre-incubation in conferring optimal cyto-protection. Such studies ascribe the prophylactic treatment’s benefits largely to potentiated antioxidant transcription, similar to observation in the current study even following radiation insult, and the kinetics of cellular uptake (Colon et al., 2010; Hirst et al., 2013; Das et al., 2018). Uptake efficiency is affected by particle properties (size, morphology, surface charging, bio-corona character), experimental conditions and cell type (Kettler et al., 2014; Singh et al., 2018). Localization of CNPs within multiple compartments (e.g., mitochondria, lysosomes, endoplasmic reticulum, nucleus) has been reported, as well as presence of a population of free particles in the cytoplasm (Singh et al., 2010). Further, sub-cellular localization has been shown to be a function of membrane transport pathway and surface charge (Asati et al., 2010; Singh et al., 2010).

The human skin is prone to photoaging due largely to UV-induced excessive production of ROS. The effect is compounded by a concomitant depletion of the endogenous antioxidant system (Shindo et al., 1994; Gęgotek et al., 2017; Oliveira et al., 2019) as a result of oxidative damage in cellular components (Photoaging et al., 2019). Our results show that pre-treatment with nanoceria decreased L929 cells UVA-induced ROS production 24 h post-irradiation; with this effect persisting even after a further dose of UVA exposure. Additionally, nanoceria was found to remain active up to 48 h. This extended ROS inhibition effect of nanoceria is attributed jointly to the catalytic nature of the particles’ enzyme-mimetic surface reactions, undergoing regenerative redox cycling (Das et al., 2013, 2014) (i.e., substantial SOD-mimetic catalytic activity ascribed to a greater presence of Ce^3+^ sites), and to modulation of endogenous antioxidant expression. These effects were further evidenced as potentiated antioxidant activity, such as SOD and GSH, through chemical assay (Singh et al., 2016). Peloi et al. (2020) demonstrated that CNPs also prevent UVB-mediated fibroblast cell oxidative damage by decreasing ROS level and increasing antioxidant enzymes activities. Other studies into nanoceria’s effect on oxidative stress conditions have evidenced changes to glutathione and SOD2 expression. Thus, these transcription level changes support the pre-incubation of nanoceria (i.e., prior to irradiation) and the observed, persistent anti-oxidant activities (Colon et al., 2010; Hirst et al., 2013; Akhtar et al., 2015).

Ultraviolet A rays radiation has been associated with most of the dermal changes in photoaged skin resulting in impaired fibroblast functions (e.g., shortening life span, inability to proliferate and senescence) (Naru et al., 2005; Nakyai et al., 2018; Lan et al., 2019). CNP contributes to the healing process by inducing migration and proliferation. The demonstrated, long-term cyto-protection and bio-activity has been previously shown for nanoceria formulations synthesized by the protocol used, herein (Hirst et al., 2009). The demonstrated wound healing activity and increased proliferation following radiation insult further confirms the improved performance of high Ce^3+^/Ce^4+^ formulations in conferring cytoprotection, across biological model systems (esp. as anti-flammation, diabetic wound healing, tumor-stroma interactions) relative to formulations evidencing fewer Ce^3+^ evolving defects (Chen et al., 2006; Chigurupati et al., 2012; Singh et al., 2016; Das et al., 2018; Shekunova et al., 2019). Findings such as these will further mediate the optimization or “nanoengineering” of CNPs toward optimal therapeutic activity in future studies.

Pretreatment with nanoceria, as well as NAC, significantly decreases UVA-induced cellular senescence, attributed to individual antioxidant activities, and thereby prevented G1 arrest (Colavitti and Finkel, 2005; Hernandez-segura et al., 2018). In agreement with previous results (Caputo et al., 2015; Li et al., 2019), we found that nanoceria decreased cell apoptosis, as assessed by caspase3/7 levels, and prevented mitochondria depolarization, as determined by TMRE labeling. It is interesting to mention that the radiation dose used here was much higher than those in the cited previous studies. This fact and the very low particle doses used suggest CNP substantial efficacy in preventing radiation damage. Preservation of mitochondrial polarization in fibroblasts was previously reported by Pezzini et al. (2017). Our results under UVA irradiation show a similar trend as those previously reported upon challenge with hydrogen peroxide. The agreement between these results support the characterization by Pezzini et al. (2017) of nanoceria as a pro-energetic agent in conditions affecting cellular redox imbalance. As UV radiation may induce DNA lesions (Rastogi et al., 2010; Fotouhi et al., 2015), protecting irradiated cells from apoptosis would imply survival of mutated cells. Caputo et al. (2015) demonstrated that CNPs did not increase micronuclei formation upon UV radiation, but almost completely prevented mutagenesis based on its antioxidant effect.

We also found that nanoceria not only inhibited L929 death but induced proliferation in both non-irradiated and UVA-irradiated cells. Nanoceria also showed the ability to stimulate wound regeneration through proliferation and migration, even after irradiation. Several studies have also shown the regenerative potential of CNPs in both cell culture and animal models (Chigurupati et al., 2012; Davan et al., 2012). The mechanism by which nanoceria induce cell proliferation is not completely understood. However, the surface chemistry dependent nature is well-represented though depends strongly, and in some cases dramatically, on particle valency, size, morphology, concentration, and exposure time (Gagnon and Fromm, 2015). The resulting cellular mechanism/response is less clear with activity attributed to activation of survival pathways such as stimulation of Bcl-2 expression and reduction of stress condition (Bizanek et al., 2018) or by nanoceria capacity in reducing apoptosis and inhibiting activation of MAP kinase pathway (Walkey et al., 2015). Interestingly, herein, we found that nanoceria increase cellular proliferation however decreased UVA-induced ERK activation. ERK1/2 phosphorylation mediates apoptosis induced by oxidative stress (Lee et al., 2003), however, the precise mechanism is unknown (Son et al., 2011). We observed in our study that UVA-induced ERK phosphorylation is closely related to the increase of ROS level and the cell survival promotion is due to the antioxidant, protective action of nanoceria.

Given the observed substantial antioxidant effects of CNPs, even at very low concentrations, under UV radiation insult, it seems reasonable that CNP-based sunscreen formulations would represent a viable strategy to protect the human skin against high doses of UV-induced damage. The viability of such a formulation is further suggested by the extended period of activity shown by the particles in conferring radio-protection.

## Conclusion

Nanoceria, even at low concentration, protect L929 fibroblast cells from oxidative damage by high doses of UVA. These particles are able to confer this cyto-protection through direct antioxidant mechanisms and play a key role in the activation of signaling pathways that control fibroblast cell aging, death, migration, and proliferation. Thus, nanoceria might be a potential ally with endogenous intracellular antioxidant enzymes to fight UVA-induced photodamage and consequently contribute to the modulation of cell lifespan. Effects on preservation of mitochondrial condition and performance, upon incubation with nanoceria, are further highlighted. Results from this and related studies demonstrate the unique character of engineered nanoceria formulations to confer cytoprotective effects under pathologic conditions and to potentiate cell growth processes of healthy cells in wound healing.

## Data Availability Statement

All datasets presented in this study are included in the article/supplementary material.

## Author Contributions

FR: methodology, investigation, data curation, writing – original draft, and writing – review and editing. MO: methodology. CVN: resources, supervision, project administration, writing – review and editing. SSi, CJN, and TS: methodology, data curation. SSe and TU-N: resources and writing – review and editing. SL: resources, supervision, project administration, and writing – review and editing. All authors contributed to the article and approved the submitted version.

## Conflict of Interest

The authors declare that the research was conducted in the absence of any commercial or financial relationships that could be construed as a potential conflict of interest.
